# Single versus double autologous stem cell transplantation and lenalidomide maintenance versus no maintenance therapy in newly-diagnosed patients with multiple myeloma: a real-life, vintage snapshot after twenty-three years from the Rete Ematologica Pugliese

**DOI:** 10.1007/s00277-025-06627-0

**Published:** 2025-10-10

**Authors:** Nicola Di Renzo, Emanuela Resta, Michelina Dargenio, Nicola Sgherza, Francesco Tarantini, Giovanni Reddiconto, Giuseppe Di Renzo, Vincenzo Federico, Maria Paola Fina, Rossella Matera, Paola Curci, Rita Rizzi, Candida Germano, Antonietta Pia Falcone, Giuseppe Mele, Rossella De Francesco, Gaetano Palumbo, Bernardo Rossini, Antonello Rana, Maria Laura Di Noi, Francesca Merchionne, Immacolata Attolico, Paola Carluccio, Nicola Cascavilla, Anna Mele, Angelo Michele Carella, Lorella Melillo, Domenico Pastore, Attilio Guarini, Vincenzo Pavone, Giorgina Specchia, Giuseppe Tarantini, Francesco Albano, Pellegrino Musto

**Affiliations:** 1 Haematology and Transplant Unit, “V. Fazzi” Hospital, Lecce, Italy; 2 Department of Clinical and Molecular Medicine, Sapienza University, Roma, Italy; 3 Hematology and Stem Cell Transplantation Unit, AOUC Policlinico, Bari, Italy; 4 Division of Hematology, Dipartimento di Medicina, “S. Maria della Misericordia” Hospital, Universita’ di Udine, Azienda Sanitaria Universitaria Friuli Centrale, Udine, Italy; 5 Department of Precision and Regenerative Medicine and Ionian Area, “Aldo Moro” University School of Medicine, Bari, Italy; 6 Hematology Unit, “Mons. Dimiccoli” Hospital, Barletta, Italy; 7 Department of Hematology and Bone Marrow Transplant, IRCSS “Casa Sollievo Della Sofferenza”, San Giovanni Rotondo, Italy; 8 Hematology Unit, “A. Perrino” Hospital, Brindisi, Italy; 9 Hematology Unit, “Cardinale Panico” Hospital, Tricase, Italy; 10 Hematology Unit, AOU Policlinico, Foggia, Italy; 11 Hematology and Cell Therapy Unit, IRCCS Istituto Tumori “Giovanni Paolo II”, Bari, Italy; 12 School of Medicine, University of Bari “Aldo Moro”, Bari, Italy

**Keywords:** Multiple myeloma, Autologous stem cell transplantation, Lenalidomide, Maintenance therapy

## Abstract

Autologous stem cell transplantation (ASCT), doubled in selected cases, followed by lenalidomide maintenance (LM) remains the standard treatment after induction therapy for newly diagnosed, transplant eligible patients with multiple myeloma (TEMM). Notwithstanding, evidences about how these approaches have been applied and how they have performed in the real-life setting, before the introduction of daratumumab within the induction regimens, are quite limited. Herein, we report the outcome of 300 MM patients, who underwent single (45%) or double (55%) ASCT, and received (42%) or not (58%) lenalidomide maintenance, outside of clinical trials, between December 2001 and February 2020, within the “Rete Ematologica Pugliese”. After a median follow-up of 65 months (range: 9-186), median PFS was significantly longer in patients who underwent double ASCT compared to those who received single ASCT (66 vs. 53 months, respectively, *p* = 0.01). Likewise, after a median follow-up of 62 months (range: 9-174), patients who received LM had a significantly better PFS respect to those who did not (72 vs. 36 months, respectively *p* < 0.001). Concerning OS, it was not influenced by single or double ASCT (although a trend favoring double ASCT was observed), while LM significantly improved OS (142 vs. 108 months, *p* = 0.01). At multivariable analysis factors influencing PFS were achievement of complete remission after first ASCT, double ASCT and LM, while those impacting on OS were high risk cytogenetics, LDH and LM. In the context of a rapidly changing therapeutic scenario, our data might contribute to a real-life, historical benchmark for current and future treatments of TEMM patients.

According to recently published 2025 EHA-EMN guidelines [1], single (or double, in selected cases) autologous stem cell transplantation (ASCT) followed by lenalidomide maintenance (LM) remains the standard of care (SOC) after induction therapy for newly diagnosed patients with multiple myeloma considered eligible for transplant procedures (TEMM). Notwithstanding, recent evidences demonstrating how these approaches have been applied and how they have performed in the real-life setting during the last years and before the introduction of the quadruplets containing anti-CD38 monoclonal antibody daratumumab within the induction regimens, are quite limited [2, 3].

Herein, we report the twenty-three years experience of eight centres belonging to the Apulian Hematological Network (Rete Ematologica Pugliese, REP) where, in particular, the clinical outcome of newly diagnosed TEMM receiving single or double ASCT, and receiving or not LM, was explored outside of clinical trials. Evaluable patients were retrospectively enrolled between December, 2001 and February, 2020, therefore in the pre-daratumumab era. The choice of treating patients with single or double ASCT and with or without LM was based according to the local policy of each centre. Primary end-points of the study were progression-free survival (PFS) and overall survival (OS). Baseline clinical characteristics and treatments received by all patients are shown in Table 1.

**Table 1:** Baseline characteristics, treatments employed and response rates, according to the number of transplant procedures and lenalidomide maintenance

	Single ASCT *n* = 135 (45%)	Double ASCT *n* = 165 (55%)	*P*	Lenalidomide maintenance *n* = 137 (48%)	No Lenalidomide maintenance *n* = 150 (52%)	*P*
Age						
Median (range)	61,8 (37–77)	58,2 (35–72)	< 0.001	62 (35–75)	60 (37–73)	ns
>65 years n. (%)	40 (30)	23 (14)	< 0.001	34 (25)	30 (20)	ns
Gender						
Males, n. (%)	73 (54)	96 (58)	ns	72 (53)	86 (57)	ns
Females, n. (%)	62 (46)	69 (42)		65 (47)	64 (43)	
ISS						
I, n. (%)	50 (37)	63 (38)	ns	60 (44)	57 (38)	ns
II, n. (%)	46 (34)	57 (35)		41 (30)	50 (33)	
III, n. (%)	39 (29)	45 (27)		36 (26)	43 (29)	
Renal function						
Serum creatinine < 2 mg/dl, n. (%)	119 (88)	149 (90)	ns	137 (100)	150 (100)	NE
Serum creatinine > 2 mg/dl, n. (%)	16 (12)	16 (10)		0 (0)	0 (0)	
LDH						
Normal, n. (%)	121 (89)	129 (85)	ns	121 (88)	129 (86)	ns
Above normal limits, n. (%)	14 (11)	23 (15)		16 (12)	21 (14)	
Cytogenetics						
Normal, n. (%)	54 (86)	59 (79)	NE	28 (90)	39 (89)	NE
High-Risk*, n. (%)	8 (14)	16 (21)		3 (10)	5 (11)	
Missing/unsuccessful, n.	73	90		106	106	
Induction type						
VTD, n. (%)	106 (78)	129 (78)	ns	106 (77)	112 (75)	ns
PAD, n. (%)	19 (14)	21 (13)		20 (15)	20 (13)	
VCD, n. (%)	10 (8)	15 (9)		11 (8)	18 (12)	
Induction cycles						
3, n. (%)	16 (12)	19 (11)	ns	15 (11)	17 (11)	ns
4, n. (%)	97 (72)	115 (70)		98 (71)	103 (69)	
5–6, n. (%)	22 (16)	31 (19)		24 (18)	30 (20)	
Mobilization						
CT +/-G-CSF, *n*. (%)	90 (67)	115 (70)	ns	95 (69)	101 (63)	ns
CT-free: G-CSF +/- plerixafor, *n*. (%)	45 (33)	50 (30)		42 (31)	49 (37)	
Conditioning melphalan dose (mg/sqm), *n*. (%)		**First ASCT**		**First ASCT**	**First ASCT**	
200	106 (79)	133 (81)	ns	105 (77)	111 (74)	ns
140	22 (16)	24 (14)		22 (16)	26 (17)	
100	7 (5)	8 (5)		10 (7)	13 (9)	
		**Second ASCT**		**Second ASCT**	**Second ASCT**	
200		20 (12)		30 (22)	30 (20)	ns
140		50 (30)		50 (36)	59 (39)	
100		95 (58)		57 (42)	61 (41)	
Number of ASCT **						
Single, n. (%)	NA	NA		63 (48)	69 (52)	ns
Double, n. (%)	NA	NA		74 (44)	81 (56)	
Response rates after ASCT						
≥ VGPR, n. (%)	119 (88)	149 (90)	ns	123 (90)	133 (89)	ns
CR + sCR, n. (%)	61 (45)	84 (51)		65 (48)	68 (52)	
Consolidation						
Yes, n. (%)	70 (52)	84 (51)	ns	70 (51)	79 (53)	ns
No, n. (%)	65 (48)	81 (49)		67 (49)	71 (47)	
Lenalidomide maintenance						
Yes, n. (%)	67 (49)	70 (51)	ns	NA	NA	
No, n. (%)	70 (47)	80 (53)				

*At least one among del(17p), t(4;14), t(14;16), del1p/gain1p21, complex karyotype. ** Thirteen patients excluded as they did not receive lenalidomide but other types of maintenance therapies. *ASCT* autologous stem cell transplantation, *CT* chemotherapy, *G-CSF* granulocyte stimulating factor, *ISS* International Staging System, *LDH* lactate dehydrogenase, *NA* not applicable, *NE* not evaluable, *ns* not significant, *VTD* (bortezomib thalidomide and dexamethasone), *PAD* (bortezomib, doxorubicin and dexamethasone), *VCD* (bortezomib, cyclophosphamide and dexamethasone), *VGPR* very good partial response, *CR* complete response, *sCR* stringent complete response

Kaplan-Meier method was used for developing OS and PFS curves. The corresponding log-rank tests were calculated and differences were estimated using the Cox model. Hazard ratios (HRs) of PFS and OS and their 95% confidence intervals (CIs) were defined using multivariable Cox proportional hazards regression.

The starting study population group consisted of 300 newly diagnosed patients with TEMM (male 56%; female 44%) who underwent single (n. 135, 45%) or, alternatively, double (n. 165, 55%) ASCT. Patients who received double ASCT had a median age lower than those who underwent single ASCT (58,2 vs. 61,8 years), as well as a smaller percentage of patients older than 65 years (14% vs. 30%, respectively). We also observed that the use of double ASCT progressively increased after 2010. No significant differences between the two groups were instead observed in terms of gender, international staging system (ISS), renal failure, lactate dehydrogenase (LDH) serum levels or cytogenetic risk at baseline. VTD (bortezomib, thalidomide and dexamethasone) was employed as induction therapy in 78% of patients, while the remaining subjects received PAD (bortezomib, doxorubicin and dexamethasone) or VCD (bortezomib, cyclophosphamide and dexamethasone) in comparable percentages. A progressive trend toward an extended use of VTD over other induction therapies was noticed after its approval in Italy.

Most patients received 4 courses of induction therapy, while approximately one third underwent 3 or up to 6 cycles in both groups. Overall response rate (ORR), at least very good partial response (≥ VGPR), and complete response (CR) plus stringent complete response (sCR) after induction were 98%, 75% and 31%, respectively, without significant differences among the different triplets utilized (data not shown). Mobilization strategies, conditioning regimens, and response rates after transplant(s), as well as percentage of patients receiving consolidation and LM were also comparable between the two groups.

At the time of analysis (October, 2024), after a median follow-up of 65 months (range: 9-186), 141 relapse/progressions were recorded (47%) and 79 patients (26%) deceased across this study population. Notably, 165 patients who had received double ASCT had a significantly longer median PFS compared to the group of 134 evaluable patients who underwent a single ASCT (66 vs. 53 months, respectively; *p* = 0.01) (Fig. 1a). A better OS was also observed for patients who received double ASCT vs. 132 evaluable patients undergoing single ASCT (median OS: 144 vs. 110 months, respectively). Such a difference, however, did not reach a statistical significance (*p* = 0.12) (Fig. 1b). Notably, no death was attributable neither to first, nor to second ASCT procedures.

**Fig. 1:**
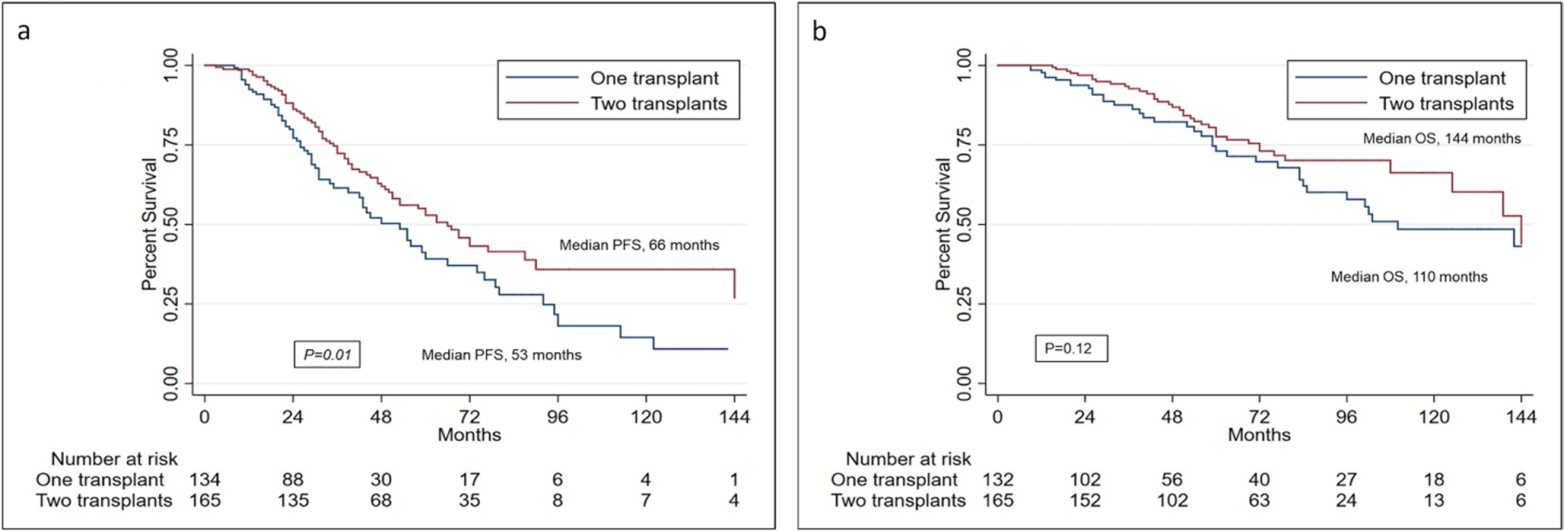
PFS (**a**) and OS (**b**), according to the number of ASCT

The role of LM after ASCT was then specifically investigated in 287 evaluable patients, selected within to the same prior cohort. Patients with progressive disease after ASCT were not included. In particular, 137 patients (42%) received LM (10 mg/d for 21–28 days, every 4 weeks, administered for a median time of 19.5 months, range 1-118 months), while 150 patients (58%) did not. Main characteristics of the two groups are also reported in Table 1. Thirteen patients who had received different types of maintenance were excluded from this analysis. Again, baseline age, gender, ISS stage, renal function, LDH levels and cytogenetic risk were comparable between these two groups. Likewise, induction and conditioning regimens, number of ASCT received, number of patients undergoing consolidation and response rates after ASCT were not statistically different. With a median follow-up of 62 months (range 9-174), median PFS was significantly longer in the LM cohort (72 vs. 36 months; *P* < 0.001) (Fig. 2a). Similarly, median OS was significantly better in patients receiving LM (142 vs. 108 months; *p* = 0.01) (Fig. 2b).

**Fig. 2:**
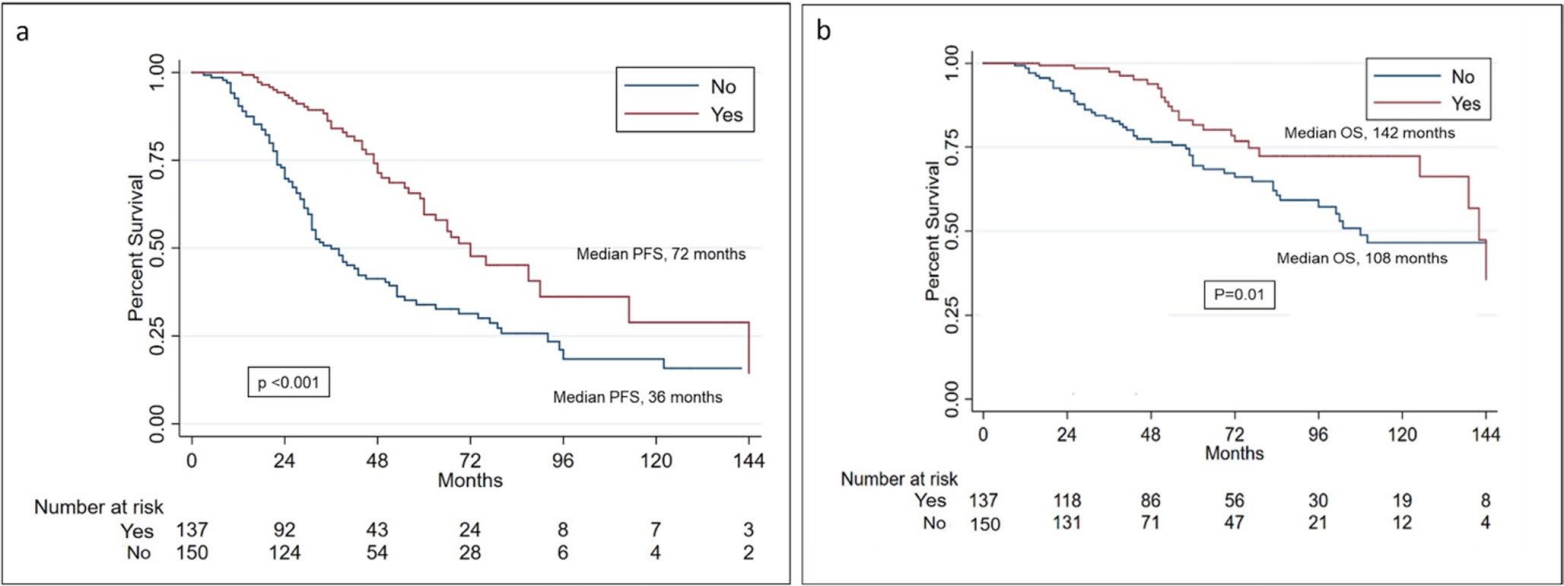
PFS (**a**) and OS (**b**), according to Lenalidomide maintenance or not

Main grade 3–4 adverse events observed in LM group were leucopenia, skin rashes and diarrhea (total 8%); they were generally managed by dose adjustment of lenalidomide and caused the interruption of maintenance in a minority of patients (6%). Three secondary primary malignancies occurred in the LM-group (one acute myeloid leukemia, one colon carcinoma and one bladder carcinoma), while 2 non-melanoma skin malignancies were observed in patients not receiving LM. There was a small fraction of responding patients (5%) that autonomously decided to interrupt LM, independently upon toxicity or inefficacy of the drug, after 18–25 months of treatment.

Univariable analysis initially included age, ISS, presence of renal failure, cytogenetic risk and LDH levels at diagnosis, type of induction treatment, number of transplants, achievement of CR (after induction, first transplant, and second transplant), consolidation therapy and LM (data not shown). Factors influencing PFS selected by multivariable analysis were achievement of CR after first ASCT (HR 0.54, CI 0.35–0.82, p 0.004), double ASCT (HR 0.50 CI 0.32–0.79, p 0.002) and LM (HR 0.60, CI 0.41–0.88, p 0.009) (Fig. 3).

**Fig. 3:**
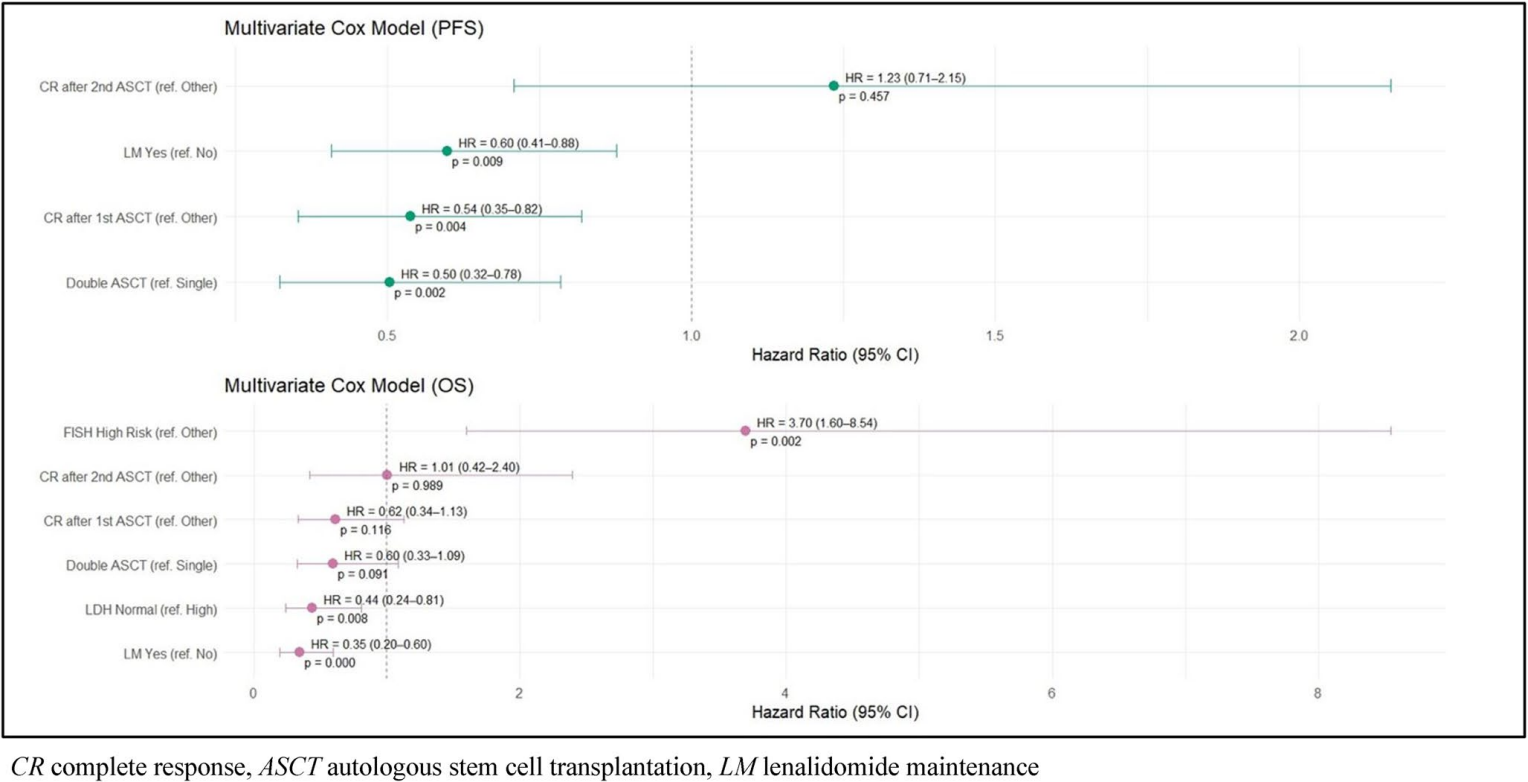
Results of multivariable analysis

Factors impacting on OS were instead high-risk cytogenetics (HR 3.70, CI 1.50–8.54, p 0.002), LDH (HR 0.44, CI 0.24–0.81, p 0.008), and LM (HR 0.35, CI 0.20-0,60, p 0.000) (Fig. 3). A not statistically significant trend favoring double ASCT was observed for OS (HR 0.60, CI 0.33–1.09, p 0.091).

While a single ASCT remains a SOC in TEMM patients [1], the role of double ASCT is still debated. Double ASCT has been particularly emphasized for patients who do not reach a deep response after a single procedure and for those showing high risk disease [1, 2, 4–9], even when modern quadruplets are employed as induction treatments [10, 11]. Interestingly, daratumumab-containing regimens followed by single ASCT, intensive consolidation and LM maintenance (with or without the addition datatumumab) in patients with high cytogenetic risk obtained lower rates of measurable residual disease (MRD)-negativity in presence of two or more high risk abnormalities. This finding suggests the need of additional strategies for these “ultra”-high risk patients [12].

In our real-life series, double ASCT significantly improved PFS, along with achievement of CR after first ASCT and LM therapy. Such a benefit, however, did not clearly emerge in terms of OS, though a not statistically significant trend was observed. High cytogenetic risk, LDH levels and, again, LM instead maintained an independent effect on OS. It should be taken into account that our patients were not selected on the basis of their risk and proceeded to single or double ASCT according to local policies. On the other hand, the reduced dose of melphalan frequently utilized for second ASCT and subsequent effective salvage treatments available in the last years may have played a role. Other studies have failed to demonstrate a favorable impact of double versus single ASCT on clinical outcome [13, 14] or MRD-achievement [15] in TEMM patients, thus further questioning the role of double ASCT. In some of these trials, however, higher dropout rates before the second ASCT and different design of the studies (i.e. prolonged induction therapies) could have represented confounding factors. Notably, improvement of PFS, but not of OS, has been also reported in TEMM patients treated with intensive VRD-based (bortezomib, lenalidomide and dexamethasone) therapy, receiving single ASCT vs. no ASCT [16].

In our study LM confirmed its tolerability, safety and, above all, efficacy [17, 18], demonstrating the capacity to double PFS and to improve significantly OS of newly diagnosed TEMM in a long-term real-life setting. Duration of LM has been specifically investigated. Studies suggest that continuing LM beyond 3 years is associated with improved PFS, while between 4 and 5 years the impact diminishes and LM could be therefore discontinued. A possible exception could be patients with still MRD-positive disease, where LM should be instead continued until it is tolerated or a relapse occurs [18, 19]. Unfortunately, regarding this aspect, our findings are not informative, as MRD data, recently recognized by FDA as a surrogate endpoint of outcome in patients with multiple myeloma, were not available.

Naturally, this study has other limits. The most important are its retrospective nature, the choice to perform one or two ASCT and LM mainly based on single centre experience/feeling and the reduced availability of cytogenetic data. Furthermore, there is no information concerning other well-known prognostic factors, such as extramedullary disease and circulating plasma cells. Thus, our findings underscore the need for more standardized therapeutic approaches for real-world TEMM patients. In this setting, future initiatives aimed at harmonizing criteria for decision-making, a systematic collection of MRD data and a comprehensive biological profiling, will permit more personalized and evidence-based treatment strategies.

The therapeutic scenario of MM is rapidly changing. Regarding TEMM patients, the use of novel quadruplets with new generation drugs as induction regimens [20], as well as sustained MRD-driven maintenance therapies based on double or even triple combinations [21], will likely represent soon new paradigms. We think our data could contribute to a real-life, historical benchmark for current and, when they will be available in the clinical practice, future treatments.

## Data Availability

No datasets were generated or analysed during the current study.
